# Long-term citrate treatment in high-risk kidney stone formers is not associated with metabolic adverse effects

**DOI:** 10.1093/ckj/sfag058

**Published:** 2026-03-09

**Authors:** Alexander Ritter, Lea Bührer, Daniel G Fuster, Nasser A Dhayat, Olivier Bonny, Gregoire Wuerzner, Thomas Ernandez, Florian Buchkremer, Stephan Segerer, Laura Keller, Maximilian Sabev, Daniel S Engeler, Beat Roth, Pietro M Ferraro, Ulrike Held, Nilufar Mohebbi, Carsten A Wagner, Harald Seeger

**Affiliations:** Division of Nephrology, University Hospital Zurich, Zurich, Switzerland; Division of Nephrology and Transplantation Medicine, HOCH Cantonal Hospital St Gallen, St Gallen, Switzerland; Department of Biostatistics and Epidemiology, Biostatistics and Prevention Institute, University of Zurich, Zurich, Switzerland; Centre for Computational Health, Institute of Computational Life Sciences, Zurich University of Applied Sciences (ZHAW), Wädenswil, Switzerland; Department of Nephrology and Hypertension, Inselspital, Bern University Hospital, University of Bern, Switzerland; B. Braun Medical Care AG, Nephrology & Dialysis Care Center, Hochfelden, Zürich, Switzerland; Service of Nephrology and Hypertension, Lausanne University Hospital and University of Lausanne, Lausanne, Switzerland; National Center of Competence of Research (NCCR) Kidney.CH; Service of Nephrology and Hypertension, Lausanne University Hospital and University of Lausanne, Lausanne, Switzerland; Division of Nephrology and Hypertension, University Hospital Geneva, Geneva, Switzerland; Division of Nephrology, Cantonal Hospital Aarau, Aarau, Switzerland; Division of Nephrology, Cantonal Hospital Aarau, Aarau, Switzerland; Division of Nephrology, University Hospital Zurich, Zurich, Switzerland; Division of Nephrology, University Hospital Zurich, Zurich, Switzerland; Division of Urology, HOCH Cantonal Hospital St Gallen, St Gallen, Switzerland; Department of Urology, Inselspital, Bern University Hospital, University of Bern, Switzerland; Division of Nephrology, University of Verona, Verona, Italy; Department of Biostatistics and Epidemiology, Biostatistics and Prevention Institute, University of Zurich, Zurich, Switzerland; Division of Nephrology, University Hospital Zurich, Zurich, Switzerland; Praxis und Dialysezentrum Zürich-City AG, Zurich, Switzerland; National Center of Competence of Research (NCCR) Kidney.CH; Institute of Physiology, University of Zurich, Zurich, Switzerland; Division of Nephrology, University Hospital Zurich, Zurich, Switzerland; Institute for Nephrology and Dialysis, Cantonal Hospital Baden, Baden, Switzerland

**Keywords:** body-mass index, glucose, lipids, stone recurrence, urine supersaturation

## Abstract

**Background:**

Citrate is frequently applied in kidney stone formers (KSFs), yet long-term safety data are lacking. We evaluated the effects of prolonged citrate therapy on metabolic health, urinary risk factors and stone recurrence in high-risk KSFs in Switzerland.

**Methods:**

The Swiss Kidney Stone Cohort (SKSC) is a multicenter study including KSFs and controls. Blood and urine analyses were performed at baseline and longitudinally over 2 years in KSFs, with subsequent telephone follow-up for stone events. A total of 654 KSFs (110 with citrate, 544 without) and 207 controls were included. Outcomes comprised anthropometric indices (body mass index, body roundness index, waist-to-hip ratio), metabolic parameters, urinary relative supersaturation ratios (RSR), stone recurrence and stone composition.

**Results:**

No evidence for between-group differences in 1- to 2-year changes in anthropometric, glucose or lipid outcomes was identified. Anthropometric indices remained stable in both groups. HbA1c rose in non-citrate (NC) but not in citrate (C) group patients. High-density lipoprotein (HDL) cholesterol increased in both groups, while low-density lipoprotein (LDL) decreased only in C patients. Propensity score–matched analyses showed no between-group differences in 1- to 2-year changes in anthropometric, glucose or lipid outcomes, with only modest within-group changes in the C group (hemoglobin A1c, HDL- and LDL-cholesterol). Urine analyses showed a greater reduction in RSR for brushite among NC patients, whereas C patients had a stronger decline in uric acid (UA) RSR. Calcium oxalate RSR decreased similarly across groups. Stone recurrence was more frequent in C patients, with 43% versus 30% of NC patients changing stone type during follow-up. No shift toward calcium phosphate stones was observed in citrate users.

**Conclusions:**

Long-term citrate therapy appeared metabolically safe, and selectively reduced UA supersaturation, while non-treated patients showed a more pronounced decrease for brushite. Higher recurrence among treated patients may reflect different baseline risk. A prospective trial is warranted to clarify additive benefits of citrate beyond dietary-guided counseling.

## Graphical Abstract

**Graphical Abstract:**
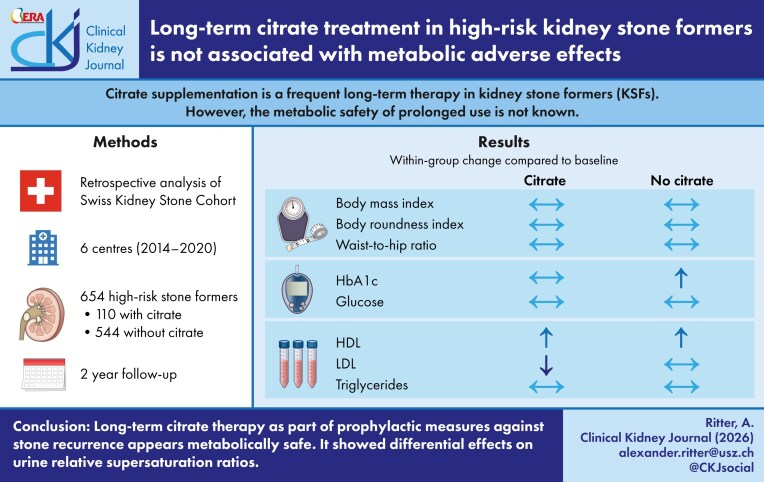

KEY LEARNING POINTS **What was known:**

- Oral citrate treatment is a key element of kidney stone prevention, increasing urinary citrate and pH, but requires caution in calcium phosphate stone formers.
- Endogenous citrate negatively regulates glycolysis and promotes lipogenesis, raising questions about its metabolic safety when used as long-term therapy.
- Existing studies demonstrated improved urinary risk profiles but only one study, limited to 3-month follow-up, examined effects on glucose and lipid metabolism without changes in glucose, hemoglobin A1c (HbA1c), cholesterol or body mass index, indicating short-term metabolic safety but leaving long-term risks unaddressed.

**This study adds:**

- No signals for metabolic risk from citrate therapy were identified in this long-term Swiss observational study. Anthropometric indices remained stable in all patients. HbA1c rose only in non-treated individuals, while low-density lipoprotein cholesterol decreased exclusively in citrate-treated patients.
- Citrate therapy reduced urine uric acid relative supersaturation ratio (RSR), whereas non-treated patients showed a greater decline in brushite RSR; calcium oxalate reduction was similar across groups.
- Despite more frequent stone recurrence and stone type changes in citrate users, no shift toward calcium phosphate stones was observed.

**Potential impact:**

- The absence of adverse metabolic signals supports the safe use of potassium citrate in long-term stone prevention strategies.
- Distinct effects on urinary RSR highlight the need for individualized therapy.
- Observed recurrence patterns underscore the importance of ongoing monitoring and tailored prevention to optimize outcomes in stone-forming patients.

## INTRODUCTION

Kidney stones are highly prevalent, with a lifetime risk of approximately 10% and a 5-year recurrence rate of 30%–40% [1, 2]. Apart from its morbidity and association with chronic kidney and cardiovascular disease, the economic burden of nephrolithiasis is high [3–5]. Addressing known risk factors is essential for prevention. Hypocitraturia and hypercalciuria are among the most important risk factors. About 80% of all kidney stones contain calcium oxalate (CaOx) or phosphate followed by uric acid (UA). Citrate can inhibit crystal formation by complexing calcium ions and raising urine pH [6]. Its supplementation has been used for recurrence prevention in kidney stone formers (KSF) with hypo- and normocitraturia and for urinary alkalinization in UA, CaOx and cystine KSFs [1, 7].

Excess weight and glucose intolerance are associated with reduced urinary citrate excretion and increased risk of kidney stones [8]. Since potassium citrate is used long-term for kidney stone prevention its metabolic safety is crucial. Endogenous citrate regulates glycolysis negatively and lipogenesis positively. The liver metabolizes some of the administered citrate to acetyl coenzyme A (acetyl-CoA), a precursor for fatty acid and cholesterol synthesis [9].

Despite its extensive prescription for stone prevention, only a short-term evaluation of citrate therapy assessing glucose and lipid metabolism over 3 months exists [10]. After 3 months, a favorable alteration in the urinary risk profile was observed, while fasting glucose, hemoglobin A1c (HbA1c), cholesterol levels and body mass index (BMI) remained unchanged, indicating short-term safety. However, this short follow-up (FU) may be insufficient to capture relevant changes in parameters such as BMI, body roundness index (BRI), waist-to-hip ratio (WHR), HbA1c and lipid profile.

This study investigated the long-term impact of citrate on the metabolic status, reflected by changes in glucose and lipid metabolism and associated anthropometric indices of high-risk KSFs in Switzerland. Additionally, we studied long-term changes in the urinary risk profile, including EQUIL2 scores [urine relative supersaturation ratios (RSR), which indicate the level by which CaOx, calcium phosphate (CaP) and UA exceed the urine equilibrium solubility], stone recurrence and composition.

## MATERIALS AND METHODS

### Study design and study population

This observational study is a retrospective analysis of prospectively collected data from the Swiss Kidney Stone Cohort (SKSC; https://sksc.nccr-kidney.ch/; NCT01990027) [11]. Participants were recruited at six centres (Aarau, Basel, Bern, Geneva, Lausanne, Zurich) between April 2014 and March 2020. The Cantonal Ethics Committee Zurich and all local committees (BASEC-ID 2022-00997) approved the study which was conducted in accordance with the Declaration of Helsinki, ICH-GCP, GEP and Swiss law. For KSF and control group eligibility and study schedule see Supplementary data, Table S1 and Ritter *et al*. [12]. The citrate group (C) is defined in Supplementary data, Table S1 and Fig. S1.

### Laboratory analyses

Blood and urine analyses were performed centrally at Bioanalytica, Lucerne, and the Department of Clinical Chemistry, Inselspital Bern. Fasting blood samples were used. Estimated glomerular filtration rate (eGFR) was calculated using the 2009 Chronic Kidney Disease Epidemiology Collaboration formula [13]. Patients collected two 24-h urine samples before visits: one preserved under oil with thymol, one native. Urine RSR were calculated using EQUIL2 software [14].

### Definitions

A stone event or recurrence was defined as one stone passage, including multiple colics or procedures for the same stone. Stones were classified by initial composition (Supplementary data, Table S2). For comparative reasons, this study uses the same definitions for 24-h urinary excretions as in the study by Wiegand *et al*. [10] (Supplementary data, Table S2). Medications were categorized as citrate therapy according to ATC Codes (Supplementary data, Table S2).

### Statistical analyses

Descriptive statistics, stratified by group, encompass mean and standard deviation for continuous variables, median and interquartile range for skewed variables, and frequencies for categorical variables. The standardized mean difference (SMD) and the Wilcoxon test quantify intergroup differences, with SMD values <0.2 indicating balance between the subgroups. Temporal variations in parameters are shown using boxplots overlayed with the individual data points, presenting measurements at baseline, 1- and 2-year FU, alongside results from the non-parametric Wilcoxon Rank Sum Test for unpaired data. Temporal correlation of parameters within groups is illustrated with scatterplots and quantified using the Pearson correlation coefficient. Kidney stone incidence per 100 patients was calculated per subgroup, and the time to the first kidney stone occurrence per patient was analysed using Kaplan–Meier estimates.

Furthermore, propensity score matching (method = “optimal”) with a 1:1 ratio has been applied for metabolic outcomes using age, sex, BMI, history of hypertension (binary), diabetes (binary), dyslipidemia (binary), gout (binary), stone composition (binary: UA/other), HbA1c (numeric) and urine citrate (numeric). Only observations with complete information on all of these variables were used for matching. All *P*-values are considered exploratory, no adjustment for multiple testing was done, and no level of significance was set. We used the R programming language, version 4.4.2 [15] in RStudio [16], integrated with dynamic programming via knitr [17].

## RESULTS

### Study population and characteristics

Of 763 SKSC participants, 654 KSFs [544 without citrate (NC), 110 with citrate (C) and 207 controls (K) were included; Supplementary data, Fig. S1]. Demographic and medical characteristics are shown in Tables 1 and 2. KSFs were older, had higher BMI, BRI and WHR, and included more men (68.3% vs 55.6%). Hypertension and diabetes were more common in KSFs, whereas vegetarian/vegan diet was less frequent. The median dose was 3400 mg (3240–6480) potassium citrate (*n* = 95), ∼33 mEq citrate, or 1863 mg (1863–1863) magnesium citrate (*n* = 16), ∼25 mEq citrate. Within KSFs, NC patients were younger (47.3 vs 52.8 years), had an earlier first stone event (36.0 vs 41.0 years) and more often a positive family history (51.5% vs 38.2%) than C patients. Hypertension was more common in C patients. NC patients more often had CaOx stones (56.7% vs 30.8%), whereas UA stones predominated in C patients (26.2% vs 3.5%). HbA1c, low-density lipoprotein (LDL) cholesterol, triglycerides, calcium and parathyroid hormone were higher in KSFs, while high-density lipoprotein (HDL) cholesterol, 25-hydroxy-vitamin D and serum bicarbonate were lower compared with controls. Controls also differed across all 24-h urine parameters. Supersaturation ratios (EQUIL2) were markedly higher in KSFs for CaOx, brushite and UA. Within KSFs, C patients had lower eGFR (89.0 vs 93.0 mL/min), lower 1,25-dihydroxy-vitamin D (98.0 vs 111.0 pmol/L), and reduced urinary pH, citrate and calcium, but higher sulfate excretion than NC patients. Supersaturation differed: brushite (RSRBr) was higher in NC patients, whereas RSRUA was higher in C patients. We found no evidence that RSRCaOx differed.

**Table 1:** Baseline characteristics of KSFs comprising the citrate (C) and non-citrate (NC) group compared with the control group (K).

Variable	Overall	C + NC	K	*P*
*n*	861	654	207	
Demographic and clinical parameters				
Age, years^a^	46.8 (36.1, 56.9)	47.9 (37.0, 58.1)	42.1 (32.0, 53.2)	<.001
Sex, male (%)^a^	562 (65.3)	447 (68.3)	115 (55.6)	.001
BMI, kg/m^2^, median (IQR)^a^	25.5 (22.9, 29.1)	26.0 (23.3, 29.9)	24.3 (22.1, 27.4)	<.001
BRI, median (IQR)^a^	4.0 (3.2, 5.6)	4.3 (3.4, 5.8)	3.3 (2.6, 4.2)	<.001
Waist circumference, cm, median (IQR)^a^	93.7 (84.0, 103.0)	96.0 (85.5, 105.0)	87.0 (78.0, 96.0)	<.001
Hip circumference, cm, median (IQR)	102.0 (97.0, 110.0)	102.0 (97.0, 110.0)	102.0 (96.0, 108.0)	.261
WHR, median (IQR)^a^	0.9 (0.8, 1.0)	0.9 (0.9, 1.0)	0.9 (0.8, 0.9)	<.001
Age at first kidney stone event, years, median (IQR)^b^	37.0 (27.0, 47.0)	37.0 (27.0, 47.0)	NA (NA, NA)	NA
Number of stone events, *n*, median (IQR)^a^	1.0 (1.0, 3.0)	2.0 (1.0, 3.0)	0.0 (0.0, 0.0)	<.001
Family history for kidney stones, *n* (%)^a^	353 (41.3)	322 (49.2)	31 (15.5)	<.001
Vegetarian/vegan diet, *n* (%)^a^	33 (3.8)	16 (2.4)	17 (8.3)	<.001
History of arterial hypertension, *n* (%)^a^	188 (21.9)	169 (25.8)	19 (9.3)	<.001
History of diabetes, *n* (%)^a^	67 (7.8)	64 (9.8)	3 (1.5)	<.001
History of dyslipidemia, *n* (%)	131 (15.3)	107 (16.4)	24 (11.8)	.138
History of gout, *n* (%)^a^	23 (2.7)	21 (3.2)	2 (1.0)	.141
Smoking (active), *n* (%)^a^	217 (25.3)	171 (26.1)	46 (22.7)	.125
Smoking in the past, *n* (%)	91 (10.6)	62 (9.5)	29 (14.3)	
Inflammatory bowel disease, *n* (%)^a^	27 (3.2)	25 (3.8)	2 (1.0)	.075
Blood parameters				
Creatinine, µmol/L, median (IQR)	76.0 (65.0, 87.0)	76.0 (65.0, 88.0)	76.0 (66.0, 85.0)	.566
eGFR, mL/min/1.73 m^2^, median (IQR)	94.0 (84.0, 100.0)	91.0 (83.0, 100.0)	100.0 (85.8, 100.0)	.389
Glucose (fasting), mmol/L, median (IQR)	5.2 (4.8, 5.7)	5.2 (4.8, 5.8)	5.2 (4.9, 5.6)	.583
HbA1c, %, median (IQR)^a^	5.3 (5.1, 5.6)	5.3 (5.1, 5.7)	5.3 (5.0, 5.5)	.033
Cholesterol (total), mmol/L, median (IQR)	4.8 (4.2, 5.6)	4.9 (4.2, 5.6)	4.7 (4.1, 5.4)	.101
LDL-cholesterol, mmol/L, median (IQR)	3.1 (2.6, 3.7)	3.2 (2.7, 3.7)	3.0 (2.4, 3.8)	.041
HDL-cholesterol, mmol/L, median (IQR)^a^	1.3 (1.0, 1.4)	1.2 (1.0, 1.4)	1.4 (1.2, 1.6)	<.001
Trigylcerides, mmol/L, median (IQR)^a^	1.1 (0.7, 1.8)	1.3 (0.7, 2.1)	1.0 (0.8, 1.4)	<.001
Calcium (total), mmol/L, median (IQR)^a^	2.3 (2.3, 2.4)	2.3 (2.3, 2.4)	2.3 (2.3, 2.4)	.008
Phosphate, mmol/L, median (IQR)	1.0 (0.9, 1.1)	1.0 (0.9, 1.1)	1.0 (0.9, 1.1)	.170
25-hydroxy-vitamin D, nmol/L, median (IQR)^a^	52.1 (34.7, 71.0)	50.8 (33.0, 70.0)	58.0 (40.0, 75.0)	.001
1,25-dihydroxy-vitamin D, pmol/L, median (IQR)	110.0 (86.0, 138.0)	109.0 (84.0, 137.8)	113.0 (92.5, 139.0)	.076
Parathormone, ng/L, median (IQR)^a^	37.5 (29.7, 48.5)	38.5 (30.5, 49.6)	35.0 (27.8, 44.0)	.001
Bicarbonate, mmol/L, median (IQR)^a^	25.0 (23.0, 27.0)	25.3 (23.2, 27.9)	23.5 (22.0, 25.0)	<.001
Bicarbonate >28 mmol/L, *n* (%)^a^	101 (11.7)	100 (15.3)	1 (0.5)	<.001
Potassium, mmol/L, median (IQR)^a^	4.1 (3.9, 4.3)	4.1 (3.9, 4.3)	4.0 (3.8, 4.1)	<.001
Potassium >5.5 mmol/L, *n* (%)	1 (0.1)	1 (0.2)	0 (0.0)	1
Chloride, mmol/L, median (IQR)^a^	103.0 (102.0, 105.0)	103.0 (101.0, 105.0)	104.0 (103.0, 106.0)	<.001
Sodium, mmol/L, median (IQR)	141.0 (140.0, 143.0)	141.0 (140.0, 143.0)	141.0 (140.0, 143.0)	.516
24-h urine parameters, median (IQR)				
Volume, L/day^a^	1.8 (1.3, 2.4)	1.8 (1.3, 2.3)	2.0 (1.5, 2.6)	<.001
pH^a^	5.9 (5.5, 6.3)	5.9 (5.4, 6.3)	6.0 (5.7, 6.4)	<.001
Creatinine, mmol/day^a^	15.8 (11.6, 20.5)	17.1 (12.6, 21.8)	12.4 (9.7, 15.6)	<.001
Sodium, mmol/day^a^	173.4 (103.6, 247.8)	208.3 (154.1, 267.8)	75.1 (58.3, 98.9)	<.001
Potassium, mmol/day^a^	75.0 (58.1, 97.2)	77.2 (60.0, 100.9)	69.8 (52.6, 86.2)	<.001
Calcium, mmol/day^a^	6.2 (4.0, 9.0)	7.1 (4.6, 9.9)	4.2 (2.7, 5.9)	<.001
Phosphate, mmol/day^a^	33.0 (23.7, 44.1)	35.4 (25.7, 48.3)	27.0 (20.0, 33.7)	<.001
Magnesium, mmol/day^a^	4.5 (3.4, 6.1)	4.8 (3.6, 6.5)	3.8 (3.1, 4.9)	<.001
Citrate, mmol/day^a^	3.5 (2.4, 4.6)	3.6 (2.4, 4.9)	3.3 (2.5, 3.9)	.006
Oxalate, mmol/day	0.3 (0.2, 0.5)	0.3 (0.2, 0.5)	0.4 (0.3, 0.5)	.030
UA, mmol/day^a^	3.7 (2.7, 4.8)	3.9 (3.0, 5.2)	3.0 (2.4, 3.8)	<.001
Sulfate, mmol/day^a^	26.7 (16.7, 32.4)	26.7 (18.8, 33.8)	18.9 (14.8, 23.8)	<.001
Chloride, mmol/day^a^	170.9 (121.0, 233.9)	190.8 (135.9, 252.7)	128.9 (97.1, 165.5)	<.001
Ammonium, mmol/day^a^	32.7 (22.3, 48.4)	35.0 (23.6, 52.8)	28.1 (20.1, 35.4)	<.001
Urine RSR, median (IQR)				
Urine RSR, CaOx^a^	6.9 (4.2, 11.1)	7.6 (4.5, 12.2)	5.4 (3.6, 8.3)	<.001
Urine RSR, calcium phosphate^a^	1.6 (0.8, 3.8)	1.8 (0.9, 4.7)	1.1 (0.5, 1.9)	<.001
Urine RSR, UA^a^	1.5 (0.7, 3.4)	2.0 (0.8, 4.0)	0.9 (0.5, 1.7)	<.001

Unit of analysis: person.

^a^SMD >0.15.

^b^indicates missingness >20%. IQR, interquartile range; NA, not available. See Supplementary data, Table S4 for exact SMD values and % missing values.

**Table 2:** Baseline characteristics of KSFs with citrate supplementation (C) compared with patients without supplementation (NC).

Variable	Overall	C	NC	*P*
*n*	654	110	544	
Demographic and clinical parameters				
Age, years, median (IQR)^b^	47.9 (37.0, 58.1)	52.8 (40.3, 62.5)	47.3 (36.4, 56.9)	.002
Sex, male (%)	447 (68.3)	80 (72.7)	367 (67.5)	.332
BMI, kg/m^2^, median (IQR)	26.0 (23.3, 29.9)	26.8 (23.6, 30.5)	25.8 (23.0, 29.8)	.230
BRI, median (IQR)	4.3 (3.4, 5.8)	4.6 (3.5, 5.8)	4.3 (3.3, 5.8)	.265
Waist circumference, cm, median (IQR)	96.0 (85.5, 105.0)	96.0 (85.0, 105.0)	95.5 (86.0, 104.2)	.716
Hip circumference, cm, median (IQR)	102.0 (97.0, 110.0)	103.0 (99.0, 110.8)	102.0 (97.0, 110.0)	.289
WHR, median (IQR)	0.9 (0.9, 1.0)	0.9 (0.9, 1.0)	0.9 (0.9, 1.0)	.630
Age at first kidney stone event, years, median (IQR)^b^	37.0 (27.0, 47.0)	41.0 (29.0, 54.0)	36.0 (26.0, 45.0)	.002
Number of stone events, *n*, median (IQR)^b^	2.0 (1.0, 3.0)	2.0 (1.0, 3.0)	2.0 (1.0, 3.0)	.186
Family history for kidney stones, *n* (%)^b^	322 (49.2)	42 (38.2)	280 (51.5)	.015
Vegetarian/vegan diet, *n* (%)	16 (2.4)	3 (2.7)	13 (2.4)	1.000
History of arterial hypertension, *n* (%)^b^	169 (25.8)	8 (43.6)	121 (22.2)	<.001
History of diabetes, *n* (%)	64 (9.8)	14 (12.7)	50 (9.2)	.336
History of dyslipidemia, *n* (%)^b^	107 (16.4)	25 (22.7)	82 (15.1)	.066
History of gout, *n* (%)^b^	21 (3.2)	7 (6.4)	14 (2.6)	.078
Smoking (active), *n* (%)	171 (26.1)	27 (24.5)	144 (26.5)	.890
Smoking in the past, *n* (%)	62 (9.5)	10 (9.1)	52 (9.6)	
Inflammatory bowel disease, *n* (%)	25 (3.8)	4 (3.6)	21 (3.9)	1.000
Stone composition, *n* (%)^b^				<.001
Struvite	9 (1.4)	4 (3.7)	5 (1.0)	
UA	46 (7.4)	28 (26.2)	18 (3.5)	
Brushite	18 (2.9)	2 (1.9)	16 (3.1)	
Apatite	24 (3.8)	3 (2.8)	21 (4.1)	
CaOx	326 (52.2)	33 (30.8)	293 (56.7)	
Other	2 (0.3)	1 (0.9)	1 (0.2)	
Mixed	6 (1.0)	0 (0.0)	6 (1.2)	
Unknown	193 (30.9)	36 (33.6)	157 (30.4)	
Time since last event, days, mean (SD)^b^	368.6 (1143.0)	566.7 (2039.0)	327.6 (844.8)	.049
Blood parameters				
Creatinine, µmol/L, median (IQR)^b^	76.0 (65.0, 88.0)	82.0 (70.0, 93.8)	75.0 (64.0, 86.2)	.001
eGFR, mL/min/1.73 m^2^, median (IQR)^b^	91.0 (83.0, 100.0)	89.0 (74.0, 100.0)	93.0 (85.0, 100.0)	<.001
Glucose (fasting), mmol/L, median (IQR)	5.2 (4.8, 5.8)	5.3 (4.8, 5.8)	5.2 (4.8, 5.8)	.496
HbA1c, %, median (IQR)	5.3 (5.1, 5.7)	5.3 (5.1, 5.7)	5.3 (5.1, 5.6)	.576
Cholesterol (total), mmol/L, median (IQR)	4.9 (4.2, 5.6)	4.9 (4.1, 5.7)	4.9 (4.2, 5.6)	.867
LDL-cholesterol, mmol/L, median (IQR)	3.2 (2.7, 3.7)	3.3 (3.0, 3.7)	3.2 (2.7, 3.7)	.336
HDL-cholesterol, mmol/L, median (IQR)	1.2 (1.0, 1.4)	1.2 (1.0, 1.3)	1.2 (1.0, 1.4)	.604
Trigylcerides, mmol/L, median (IQR)^b^	1.3 (0.7, 2.1)	1.6 (0.8, 3.0)	1.2 (0.7, 2.0)	.006
Calcium (total), mmol/L, median (IQR)	2.3 (2.3, 2.4)	2.3 (2.3, 2.4)	2.3 (2.3, 2.4)	.829
Phosphate, mmol/L, median (IQR)	1.0 (0.9, 1.1)	1.0 (0.8, 1.1)	1.0 (0.9, 1.1)	.908
25-hydroxy-vitamin D, nmol/L, median (IQR)	50.8 (33.0, 70.0)	50.8 (33.0, 72.0)	50.9 (33.0, 69.8)	.754
1,25-dihydroxy-vitamin D, pmol/L, median (IQR)^b^	109.0 (84.0, 137.8)	98.0 (74.0, 124.0)	111.0 (86.0, 140.0)	<.001
Parathormone, ng/L, median (IQR)^b^	38.5 (30.5, 49.6)	36.2 (28.7, 50.0)	39.0 (30.6, 49.6)	.152
Bicarbonate, mmol/L, median (IQR)	25.3 (23.2, 27.9)	25.4 (23.5, 27.0)	25.3 (23.2, 28.0)	.749
Bicarbonate >28 mmol/L, *n* (%)^b^	100 (15.3)	11 (10.0)	89 (16.4)	.122
Potassium, mmol/L, median (IQR)	4.1 (3.9, 4.3)	4.2 (4.0, 4.3)	4.1 (3.9, 4.3)	.258
Potassium >5.5 mmol/L, *n* (%)	1 (0.2)	1 (0.2)	0 (0.0)	1
Chloride, mmol/L, median (IQR)^b^	103.0 (101.0, 105.0)	103.0 (101.0, 105.0)	103.0 (101.0, 105.0)	.242
Sodium, mmol/L, median (IQR)^b^	141.0 (140.0, 143.0)	141.0 (140.0, 142.0)	141.0 (140.0, 143.0)	.057
Urine parameters, median (IQR)				
Volume, L/day	1.8 (1.3, 2.3)	1.8 (1.4, 2.4)	1.8 (1.2, 2.3)	.195
pH^b^	5.9 (5.4, 6.3)	5.7 (5.2, 6.1)	5.9 (5.5, 6.3)	.002
Creatinine, mmol/day	17.1 (12.6, 21.8)	17.0 (12.1, 21.9)	17.1 (12.8, 21.8)	.906
Sodium, mmol/day	208.3 (154.1, 267.8)	210.7 (152.6, 261.0)	206.9 (154.2, 272.0)	.698
Potassium, mmol/day	77.2 (60.0, 100.9)	77.9 (59.8, 100.7)	77.0 (60.0, 101.1)	.884
Calcium, mmol/day^b^	7.1 (4.6, 9.9)	5.3 (3.7, 8.0)	7.4 (4.8, 10.2)	<.001
Phosphate, mmol/day	35.4 (25.7, 48.3)	35.4 (27.9, 44.2)	35.4 (25.5, 49.4)	.661
Magnesium, mmol/day	4.8 (3.6, 6.5)	4.7 (3.4, 6.5)	4.9 (3.6, 6.5)	.523
Citrate, mmol/day^b^	3.6 (2.4, 4.9)	2.7 (1.7, 4.1)	3.8 (2.5, 5.0)	<.001
Oxalate, mmol/day^b^	0.3 (0.2, 0.5)	0.4 (0.2, 0.6)	0.3 (0.2, 0.5)	.122
UA, mmol/day	3.9 (3.0, 5.2)	4.0 (2.9, 5.1)	3.8 (3.0, 5.2)	.935
Sulfate, mmol/day^b^	26.7 (18.8, 33.8)	29.9 (19.0, 41.9)	26.7 (18.8, 32.5)	.046
Chloride, mmol/day	190.8 (135.9, 252.7)	194.3 (136.3, 246.5)	190.0 (135.7, 254.7)	.742
Ammonium, mmol/day	35.0 (23.6, 52.8)	34.2 (21.4, 57.5)	35.3 (24.3, 52.4)	.599
Urine RSR, median (IQR)				
Urine RSR, CaOx	7.6 (4.5, 12.2)	6.7 (4.3, 12.3)	7.7 (4.6, 12.2)	.182
Urine RSR, calcium phosphate	1.8 (0.9, 4.7)	1.2 (0.4, 4.1)	1.9 (0.9, 4.8)	.008
Urine RSR, UA^b^	2.0 (0.8, 4.0)	2.5 (0.9, 4.8)	1.9 (0.8, 3.8)	.031

Unit of analysis: person.

^a^SMD >0.15. IQR, interquartile range. See Supplementary data, Table S5 for exact SMD values and % missing.

### Main urinary risk factors

At baseline hypercalciuria was more frequent in NC and hypocitraturia in C patients (Table 3). Sex-specific differences are shown in Supplementary data, Table S3.

**Table 3:** Number and percentage of subjects with hypercalciuria, hypocitraturia, hyperuricosuria and hyperoxaluria at baseline, stratified by group.

Variable	Overall	C	NC	K^a^	*P*	SMD
*n*	860	110	544	206		
Hypercalciuria, *n* (%)	358 (41.6)	36 (32.7)	300 (55.1)	22 (10.7)	<.001	0.698
Hypocitraturia, *n* (%)	77 (9.0)	21 (19.1)	41 (7.5)	15 (7.3)	<.001	0.236
Hyperuricosuria, *n* (%)	229 (26.6)	33 (30.0)	169 (31.1)	27 (13.1)	<.001	0.295
Hyperoxaluria, *n* (%)	235 (27.4)	35 (31.8)	136 (25.0)	64 (31.2)	.125	0.101

^a^One patient was excluded from the K group due to lack of data regarding calciuria. Unit of analysis: person.

### Metabolic status over time

**Figure 1:**
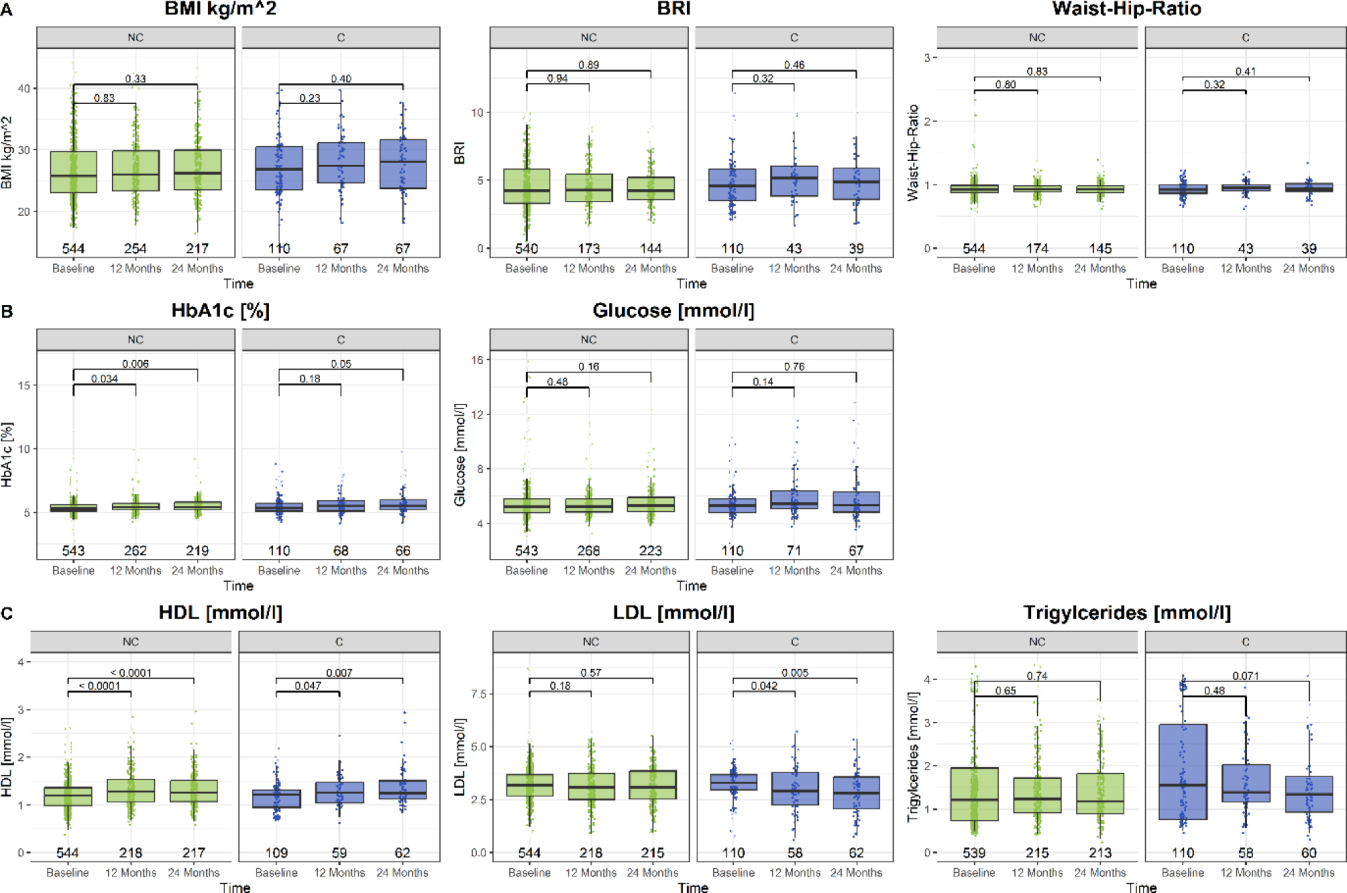
Plot of metabolic parameters for patients receiving citrate (C) and patients not receiving citrate (NC) at baseline vs 1 year and 2 years FU. (**A**) Anthropometric indices; (**B**) glucose metabolism, (**C**) lipid metabolism. The number at the bottom per boxplot refers to the number of patients included per time point and group.

Main metabolic outcomes are shown in Table 4 and Fig. 1. There were no between-group differences in changes from baseline in anthropometric indices and glucose/lipid parameters after 1 and 2 years (Table 4). In both the C and NC groups, BMI, BRI and WHR remained stable (Fig. 1A). However, HbA1c rose in NC patients at both time points but not in C patients, while fasting glucose remained unchanged (Fig. 1B). HDL-cholesterol increased in both groups, LDL decreased only in C patients, and triglycerides remained stable (Fig. 1C). New diabetes therapy was initiated in 0.9% of C (*n* = 1) and NC patients (*n* = 5). Lipid-lowering medication was started in 1.8% of C (*n* = 2) and 0.9% of NC (*n* = 5) patients. Median intact parathyroid hormone and potassium rose slightly in C and declined in NC patients (Table 4). No severe hyperkalemia (>5.5 mmol/L) occurred. At 2 years, serum bicarbonate declined less in C than in NC patients. The prevalence of bicarbonate >28 mmol/L was similar (1 year: 10.1% vs 9.2%, *P* = .997; 2 years: 6.3% vs 4.7%, *P* = .836).

**Table 4:** Change of clinical, blood and urine parameters of patients with citrate supplementation (C group) compared with patients without supplementation (NC group) between baseline and 12- and 24-month FU.

	12 months				24 months			
Variable	Mean change C	Mean change NC	*P*	SMD missing (%)	Mean change C	Mean change NC	*P*	SMD missing (%)
*n*	72	268			69	225		
BMI, kg/m^2^, median (IQR)	0.5 (–0.3, 1.1)	0.3 (–0.4, 1.0)	.628		0.2 (–0.5, 1.1)	0.4 (–0.4, 1.2)	.574	
BRI, median (IQR)	0.1 (–0.3, 0.5)	0.1 (–0.3, 0.6)	.788	^b^	–0.1 (–0.6, 0.8)	0.1 (–0.4, 0.6)	.515	^b^
Waist circumference, cm, median (IQR)	1.0 (–2.0, 4.0)	0.1 (–2.4, 5.0)	.782	^b^	0.0 (–4.5, 7.5)	1.0 (–3.0, 5.0)	.831	^b^
Hip circumference, cm, median (IQR)	0.0 (–3.0, 3.2)	0.0 (–3.0, 3.0)	.856	^b^	–1.0 (–5.2, 3.5)	1.0 (–2.0, 5.0)	.151	^a,b^
WHR, median (IQR)	0.0 (0.0, 0.0)	0.0 (0.0, 0.1)	.962	^b^	0.0 (0.0, 0.0)	0.0 (0.0, 0.0)	.931	^b^
Blood pressure systolic, mmHg, median (IQR)	–3.0 (–10.5, 7.2)	–3.2 (–11.1, 5.5)	.652		–2.0 (–10.8, 7.5)	–1.5 (–11.1, 7.0)	.396	^a^
Blood pressure diastolic, mmHg, median (IQR)	–0.5 (–7.8, 6.5)	0.0 (–6.0, 6.5)	.792		–1.0 (–8.2, 8.2)	0.5 (–5.0, 7.5)	.421	
Creatinine, µmol/L, median (IQR)	2.5 (–4.2, 7.0)	1.0 (–4.0, 6.0)	.298		4.0 (–2.0, 8.0)	1.0 (–4.0, 6.0)	.012	^a^
eGFR, mL/min/1.73 m^2^, median (IQR)	0.0 (–6.5, 2.2)	0.0 (–7.0, 1.0)	.737		–4.0 (–10.0, 0.0)	–2.0 (–9.2, 0.0)	.509	
Glucose (fasting), mmol/L, median (IQR)	0.1 (–0.3, 0.6)	0.1 (–0.3, 0.4)	.553	^a^	0.1 (–0.3, 0.4)	0.1 (–0.3, 0.5)	.826	
HbA1c, %, median (IQR)	0.0 (–0.2, 0.3)	0.1 (–0.1, 0.2)	.548		0.1 (–0.1, 0.3)	0.1 (–0.1, 0.2)	.618	^a^
Cholesterol (total), mmol/L, median (IQR)	0.0 (–0.4, 0.4)	0.0 (–0.3, 0.4)	.385		0.1 (–0.2, 0.5)	0.0 (–0.3, 0.5)	.917	
LDL-cholesterol, mmol/L, median (IQR)	0.0 (–0.6, 0.3)	0.0 (–0.6, 0.4)	.565		–0.1 (–0.9, 0.3)	0.0 (–0.5, 0.5)	.105	
HDL-cholesterol, mmol/L, median (IQR)	0.0 (–0.1, 0.2)	0.0 (–0.1, 0.2)	.673	^a^	0.1 (–0.1, 0.2)	0.1 (–0.1, 0.2)	.764	
Trigylcerides, mmol/L, median (IQR)	0.0 (–0.5, 0.4)	0.0 (–0.3, 0.4)	.516	^a^	–0.1 (–0.6, 0.4)	0.1 (–0.5, 0.4)	.176	
Calcium (total), mmol/L, median (IQR)	0.0 (–0.1, 0.1)	0.0 (–0.1, 0.1)	.646		0.0 (0.0, 0.1)	0.0 (–0.1, 0.1)	.406	
Phosphate, mmol/L, median (IQR)	0.0 (–0.1, 0.1)	0.0 (–0.1, 0.1)	.909		0.0 (–0.1, 0.1)	0.0 (–0.1, 0.1)	.612	
25-hydroxy-vitamin D, nmol/L, median (IQR)	1.1 (–7.1, 13.2)	2.0 (–7.1, 13.0)	.912		2.0 (–8.0, 13.0)	–0.6 (–11.0, 12.0)	.250	
1,25-dihydroxy-vitamin D, pmol/L, median (IQR)	10.5 (–12.5, 33.5)	2.5 (–19.0, 25.0)	.159	^a^	12.0 (–5.0, 30.0)	7.0 (–18.0, 31.0)	.335	
Parathormone, ng/L, median (IQR)	0.6 (–5.4, 7.6)	–2.7 (–9.8, 3.3)	.016	^a^	0.2 (–7.1, 6.3)	–2.1 (–9.3, 6.7)	.464	
Bicarbonate, mmol/L, median (IQR)	–0.7 (–2.0, 1.0)	–1.0 (–2.3, 1.0)	.300	^a^	–0.3 (–1.9, 0.8)	–1.2 (–2.9, 0.3)	.042	^a^
Potassium, mmol/L, median (IQR)	0.1 (–0.3, 0.2)	–0.1 (–0.3, 0.2)	.047	^a^	0.0 (–0.3, 0.2)	–0.1 (–0.3, 0.1)	.078	^a^
Chloride, mmol/L, median (IQR)	1.0 (–1.0, 2.0)	0.0 (–1.0, 2.0)	.772		–1.0 (–2.0, 1.8)	1.0 (–1.0, 3.0)	.004	^a^
Sodium, mmol/L, median (IQR)	0.0 (–1.0, 2.0)	0.0 (–2.0, 1.0)	.285		–1.0 (–2.0, 1.0)	0.0 (–2.0, 2.0)	.154	^a^
Urine parameters, median (IQR)								
Volume, L/day	0.1 (–0.2, 0.7)	0.2 (–0.1, 0.6)	.412		0.1 (–0.4, 0.6)	0.2 (–0.2, 0.6)	.299	^a^
pH	0.5 (0.0, 1.0)	0.0 (–0.3, 0.3)	<.001	^a^	0.4 (0.0, 0.8)	0.0 (–0.3, 0.4)	<.001	^a^
Creatinine, mmol/day	–3.2 (–7.5, –1.0)	–4.0 (–8.3, –0.5)	.828		–4.3 (–7.8, –0.5)	–3.7 (–8.4, –0.3)	.589	
Sodium, mmol/day	–36.9 (–85.9, 10.5)	–53.1 (–103.9, 3.0)	.219		–34.4 (–94.6, 7.1)	–49.6 (–98.3, 5.9)	.313	
Potassium, mmol/day	–3.0 (–32.7, 24.5)	–16.2 (–40.1, –0.1)	.004	^a^	0.1 (–28.4, 25.8)	–15.1 (–38.5, 2.5)	.001	^a^
Calcium, mmol/day	–1.3 (–3.7, 0.0)	–1.7 (–3.8, 0.3)	.625		–1.4 (–3.0, –0.1)	–1.4 (–4.1, 0.5)	.873	
Phosphate, mmol/day	–11.7 (–19.5, –2.5)	–8.8 (–20.8, –0.1)	.354		–8.0 (–18.4, –0.6)	–8.0 (–18.7, 0.8)	.960	
Magnesium, mmol/day	–1.2 (–2.4, –0.3)	–1.1 (–2.6, 0.0)	.776		–1.3 (–2.6, 0.0)	–0.9 (–2.4, 0.3)	.618	
Citrate, mmol/day	0.0 (–1.2, 1.2)	–0.7 (–1.7, 0.0)	<.001	^a^	0.1 (–0.9, 1.3)	–0.7 (–1.7, 0.2)	<.001	^a^
Oxalate, mmol/day	–0.1 (–0.2, 0.1)	0.0 (–0.2, 0.1)	.060	^a^	0.0 (–0.2, 0.1)	0.0 (–0.1, 0.2)	.180	
UA, mmol/day	–0.7 (–1.7, –0.1)	–0.9 (–2.1, –0.1)	.936		–1.2 (–1.9, 0.0)	–0.7 (–1.8, 0.0)	.551	
Sulfate, mmol/day	–8.1 (–21.9, 0.2)	–6.1 (–13.1, 0.2)	.169	^a^	–11.9 (–19.7, –1.2)	–7.5 (–16.6, –0.1)	.178	^a^
Chloride, mmol/day	–22.6 (–100.8, 15.1)	–49.8 (–107.4, –3.5)	.099		–31.2 (–92.9, 7.5)	–45.3 (–110.8, 1.5)	.311	^a^
Ammonium, mmol/day	–14.1 (–34.4, –2.5)	–10.1 (–27.2, 1.6)	.107	^a^	–15.0 (–29.2, –2.4)	–5.9 (–22.5, 5.1)	.014	^a^
Urine RSR, CaOx	–2.6 (–6.0, 0.6)	–1.9 (–6.1, 1.2)	.484		–2.2 (–6.7, 0.4)	–0.8 (–4.8, 1.8)	.146	
Urine RSR, calcium phosphate	0.0 (–1.6, 0.4)	–0.7 (–3.1, 0.2)	.014		–0.1 (–1.4, 0.4)	–0.6 (–3.1, 0.1)	.042	
Urine RSR, UA	–1.0 (–3.5, –0.2)	–0.4 (–1.7, 0.2)	.001	^a^	–1.4 (–3.9, –0.2)	–0.4 (–1.8, 0.3)	<.001	^a^

Unit of analysis: person with baseline and FU visit after 12 and 24 months.

^a^SMD >0.15.

^b^missingness >20%. IQR, interquartile range.

Propensity score–matched analyses for the main metabolic outcomes (Supplementary data, Fig. S2) with 107 subjects in each group (Table 5 and Supplementary data, Table S6) did not alter the absence of between-group differences in changes from baseline in anthropometric indices or glucose and lipid parameters at 1 and 2 years (Table 5 and Supplementary data, Table S7). Relative to baseline, subtle within-group changes were observed only in the C group for HbA1c (increase at 2 years), HDL-cholesterol (increase at 1 and 2 years) and LDL-cholesterol (decrease at 2 years) (Supplementary data, Fig. S3).

**Table 5:** Metabolic baseline characteristics and their change in the propensity score-matched population of patients with citrate supplementation (C group) compared with patients without supplementation (NC group) between baseline and 12- and 24-month FU.

	Baseline				12 months				24 months			
Variable	C	NC	*P*	SMD missing (%)	Mean change C	Mean change NC	*P*	SMD missing (%)	Mean change C	Mean change NC	*P*	SMD missing (%)
*n*	107	107			70	58			68	46		
Clinical parameters, median (IQR)												
BMI, kg/m^2^	26.6 (23.5, 30.5)	25.9 (23.5, 30.2)	.661		0.5 (–0.4, 1.1)	0.0 (–0.5, 0.7)	.126	^a^	0.2 (–0.6, 1.1)	0.0 (–0.7, 0.4)	.169	^a^
BRI	4.6 (3.4, 5.8)	4.1 (3.5, 5.6)	.329	^a^	0.1 (–0.3, 0.5)	0.0 (–0.3, 0.5)	.636	^b^	–0.1 (–0.6, 0.8)	0.0 (–0.6, 0.4)	.986	^b^
WHR	0.9 (0.9, 1.0)	0.9 (0.9, 1.0)	.791		0.0 (0.0, 0.0)	0.0 (0.0, 0.0)	.616	^b^	0.0 (0.0, 0.0)	0.0 (0.0, 0.0)	.632	^a,b^
Blood parameters, median (IQR)												
Glucose (fasting), mmol/L	89.0 (74.0, 100.0)	90.0 (75.5, 100.0)	.801		0.1 (–0.3, 0.6)	0.1 (–0.3, 0.3)	.624	^a^	0.1 (–0.3, 0.4)	0.2 (–0.2, 0.6)	.442	
HbA1c, %,	5.3 (4.8, 5.8)	5.5 (4.9, 6.0)	.291		0.0 (–0.2, 0.3)	0.0 (–0.1, 0.3)	.587		0.1 (–0.1, 0.3)	0.0 (–0.1, 0.3)	.556	
Cholesterol (total), mmol/L	5.3 (5.1, 5.7)	5.4 (5.1, 5.8)	.612		0.0 (–0.4, 0.4)	–0.1 (–0.4, 0.3)	.923		0.1 (–0.2, 0.5)	–0.1 (–0.4, 0.3)	.315	^a^
LDL-cholesterol, mmol/L	4.9 (4.1, 5.7)	5.0 (4.1, 5.7)	.649		0.0 (–0.6, 0.3)	–0.1 (–0.6, 0.3)	.949		–0.1 (–0.8, 0.4)	–0.1 (–0.5, 0.5)	.349	^a^
HDL-cholesterol, mmol/L	3.3 (3.0, 3.7)	3.0 (2.5, 3.7)	.007	^a^	0.0 (–0.1, 0.2)	0.0 (–0.2, 0.2)	.335		0.1 (–0.1, 0.2)	0.0 (–0.1, 0.3)	.771	
Trigylcerides, mmol/L	1.2 (0.9, 1.3)	1.3 (1.0, 1.4)	.110		0.0 (–0.6, 0.4)	0.1 (–0.3, 0.4)	.368	^a^	–0.1 (–0.6, 0.4)	–0.1 (–0.5, 0.4)	.707	

Unit of analysis: person with baseline and FU visit after 12 and 24 months.

^a^SMD >0.15.

^b^missingness >20%. IQR, interquartile range.

### Urinary risk profile over time

**Figure 2:**
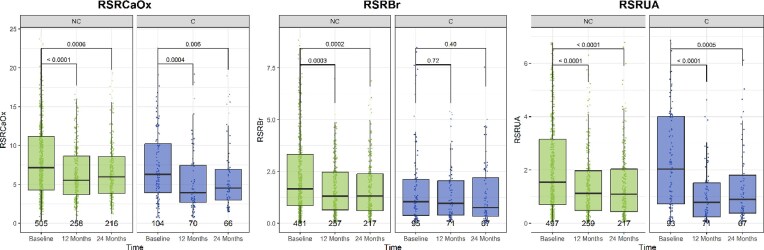
Plot of RSRCaOx, RSRBr and RSRUA for patients receiving citrate (C) and patients not receiving citrate (NC) at baseline vs 1 year and 2 years FU. The number at the bottom per boxplot refers to the number of patients included per time point and group.

Changes in RSR differed between groups after 1 and 2 years (Table 4). NC patients showed a decrease in CaP (RSRBr), whereas C patients had a more pronounced reduction in RSRUA. We found no evidence for a between-group difference for RSRCaOx. In both groups (C, NC), RSRCaOx and RSRUA decreased during FU, while in contrast to NC patients there was no evidence for a decline in RSRBr in C patients (Fig. 2). Changes from baseline in 24-h urine pH, potassium, and citrate excretion differed between C and NC groups at both 1 and 2 years (Table 4). After 2 years, urine ammonium declined more in C than NC patients. Urine volume increased in both groups at 1 year, but at 2 years remained only in NC patients (Supplementary data, Fig. S4). During FU, 24-h urine pH rose in C patients but was stable in NC. Potassium and citrate excretion remained stable in C and declined in NC. Sodium, chloride, magnesium, phosphate, sulfate and UA excretion decreased in both groups, while oxaluria remained unchanged.

### Stone events and composition over time

A higher percentage of C patients had one or more stone event after baseline compared with NC (21.2% vs 11.1%) within 7.8 years of FU. The 1-year incidence was 14.55 [confidence interval (CI) 8.91–23.74] per 100 patient-years in C and 4.96 (CI 3.40–7.24) in NC. At 2 years, rates were 10.91 (CI 7.31–16.28) and 4.32 (CI 3.25–5.75), respectively. Time-to-event analysis showed a shorter interval in the C group (*P* = .0031).

**Figure 3:**
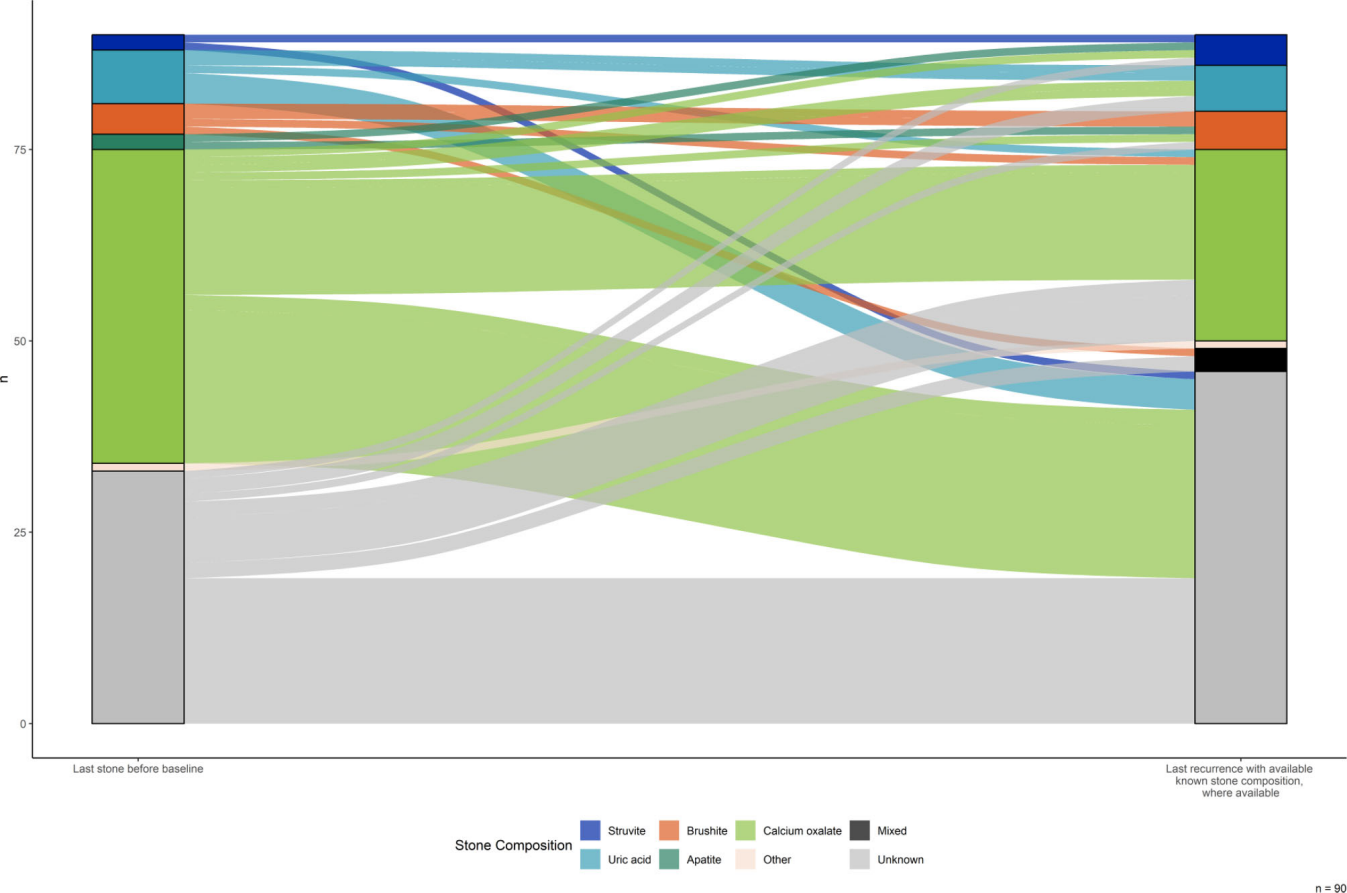
Visualization of the stone composition over time. The last stone before baseline visit and the last recurrence with available stone composition after baseline visit are illustrated. Stone composition: apatite; brushite; CaOx; mixed; other; struvite; UA; unknown.

Stone composition of patients with events (C, *n* = 28; NC, *n* = 61) is shown in Fig. 3. Stone analysis was unavailable for both events in 75.0% (*n* = 21) of C and 63.9% (*n* = 39) of NC patients, the last stone before inclusion and a stone event after inclusion. Among KSFs with known stone composition, 42.9% (*n* = 3) in the C group and 30.4% (*n* = 7) in the NC group changed stone category (Supplementary data, Table S8). In C, one patient (14.3%) shifted from CaOx to struvite, one to UA, and one from apatite to struvite. In NC, one patient (4.3%) converted from CaOx to brushite, and one to UA; conversions from UA and brushite to CaOx (each 4.3%) and from apatite to brushite (4.3%) also occurred. No UA to CaP transitions were observed.

## DISCUSSION

Although considered safe, chronic citrate treatment may have long-term side effects [10, 18]. We analysed SKSC data to address potential negative effects of citrate intake.

Citrate is a crucial tricarboxylic acid cycle intermediate and may influence numerous metabolic processes. Via fructose-1,6-bisphosphatase, citrate can stimulate gluconeogenesis [19]. Experimental data show that citrate supplementation can modulate glucose metabolism, sometimes improving glucose homeostasis, but effects are context-dependent and may vary with metabolic status [20]. Excessive citrate may disrupt normal glucose regulation, especially with insulin resistance or metabolic syndrome. Experimental studies suggest that increased citrate flux may impair insulin sensitivity, yet evidence in humans is limited [21]. Suppression of isocitrate dehydrogenase compromised glucose-stimulated insulin secretion in β-cells [19, 22]. Despite these potential metabolic properties, clinical studies have not investigated adverse long-term metabolic effects of citrate treatment [23–25]. In the SKSC, we observed a HbA1c increase within the NC group at 1 and 2 years but not in the C group, with no evidence for between group difference. Fasting glucose did not change in either group, with no difference in antidiabetic drug initiation during FU. After propensity score matching, changes of HbA1c and fasting glucose remained comparable between groups. Relative to baseline, subtle HbA1c within-group changes were observed only in the C group but this marginal increase is of questionable clinical relevance. Altogether, these findings suggest no major negative influence of citrate on glucose metabolism.

Citrate is a key precursor for cytosolic acetyl-CoA, the substrate for fatty acid and cholesterol synthesis [26]. Excessive fatty acid buildup contributes to metabolic diseases, such as diabetes and metabolic-dysfunction associated liver disease (MASLD) [27, 28]. High cytosolic citrate can activate acetyl-CoA carboxylase, stimulating lipogenesis [29, 30] via malonyl-CoA, which promotes fatty acid synthesis and inhibits β-oxidation [31]. Experimental studies indicate that exogenous citrate promotes lipid accumulation. In preclinical studies, high citrate levels activate mTOR, enhance lipogenesis and induce senescence [32]. In *Caenorhabditis elegans*, citrate supplementation triggers the mitochondrial unfolded protein response and lipid buildup [33]. Long-term high-dose citrate may drive lipogenesis, leading to fatty liver, hyperlipidemia or obesity. In our study, triglycerides (TG) did not increase. There were no negative effects on LDL- or HDL-cholesterol. No other studies so far have investigated the long-term effect of citrate on lipid metabolism. In hemodialysis patients, citrate dialysate raised LDL and decreased HDL and TG compared with acetate [34]. However, these effects were observed after 4 weeks of treatment without long-term data. In our study, lipid-lowering drug use did not increase during observation. Conclusively, citrate treatment was not associated with adverse effects on lipid metabolism over 2 years. Conversely, LDL decreased and HDL increased in the citrate group in both analyses (unadjusted, propensity score–matched). Whether this was a consequence of lifestyle recommendations at baseline or an effect of citrate cannot be distinguished. Changes in BMI, BRI and WHR at 1 and 2 years did not differ between groups and remained stable in both analyses (Fig. 1, Table 4). Furthermore, no safety concerns regarding metabolic alkalosis or hypokalemia were identified.

Citrate therapy may increase citrate excretion and lower stone risk, but may raise urine pH, thereby increasing CaP stone risk. Urine RSR or relative supersaturations (RSS) of CaOx, CaP and UA are used to estimate stone risk. RSR is calculated using software (e.g. EQUIL2) that factors in the ion activity product of a given salt and its thermodynamic solubility product while the RSS is a semi-empirical score derived from urine chemistry [14, 35]. Cross-sectional studies have demonstrated that RSS is associated with the risk for stone formation [36, 37], and in hypercalciuric KSFs undergoing two different diets, the change in CaOx RSS (EQUIL2) after 1 week predicted recurrence risk of CaOx stones [38]. In our study, RSR for all stone types were higher in stone formers compared with controls. This is in contrast to Robert *et al*., who did not find a difference in urine RSR for CaOx between KSFs and controls using EQUIL2 [39]. Changes in urine RSR varied between groups after 1 and 2 years (Table 4). The RSR of CaOx decreased at 12 and 24 months in both groups in a similar fashion. It has been demonstrated that citrate supplementation lowers supersaturation [40–43]. However, earlier studies did not use EQUIL2 to estimate supersaturation. The absence of a between-group difference in decrease could be explained by the strong reduction in calciuria at 12 and 24 months, which occurred in both groups. We would have expected a stronger calciuria decrease in the C group, as citrate treatment normally reduces calciuria [44, 45]. The marked calciuria reduction in the NC group and the lack of difference between groups may reflect the decrease in natriuria [46, 47]. Reduction in natriuria, likely resulting from nutritional counseling and reduced salt intake, was numerically more pronounced in the NC group. Consequently, the absence of a difference in RSR for CaOx between NC and C at FU may be attributable to nutritional counseling aimed at reducing sodium and animal protein intake. The decrease in RSR for brushite in the NC group and the lack thereof in the C group is consistent with the results of Doizi *et al*. [48], who reported an increase in RSRBr (EQUIL2) in brushite stone formers when citrate was initiated. We speculate that the difference in our study was driven by the increase in urinary pH in the C group due to citrate supplementation. The C group exhibited a more pronounced reduction in RSRUA at both FU timepoints, potentially driven by an increase in urine pH and stable citraturia compared with the decreasing citraturia in the NC group. Taken together, citrate supplementation in high-risk KSFs had a different impact on RSR for the stone types analysed.

A meta-analysis of seven randomized controlled trials (*n* = 324) reported a relative risk reduction of 74% in new stones with citrate [49]. In contrast, over 7.8 years, our C group had nearly twice the stone recurrence as the NC group, with markedly higher rates at 12 and 24 months, indicating substantial differences in KSF characteristics. The C group included more UA stone formers, who carry a greater recurrence risk than CaOx stones [50], as well as lower baseline citraturia and a longer mean time since the last stone event (566.7 vs 327.6 days), possibly causing inverse lead-time bias. Physicians may also have preferentially prescribed citrate to patients perceived at higher recurrence risk, possibly based on unmeasured factors such as presumed low adherence to lifestyle or dietary recommendations. Alternatively, the effect may be genuine, reflecting the repeated lifestyle and dietary counseling which likely reduced recurrence risk in both groups and may have limited the observable benefits of citrate.

Potassium citrate raises urinary citrate and pH while reducing calciuria, but excessive alkalinization may promote CaP stone formation. Previous studies suggest recurrent CaOx stone formers may shift toward CaP, specifically apatite or brushite stones [51]. Citrate supplementation could facilitate this shift. However, our analysis found no evidence for a relevant shift. There was no transition from UA or CaOx to brushite or apatite stones in the C group (Supplementary data, Table S8). Conversely, in the NC group one case shifted from CaOx to brushite, accounting for 10% of the recurrent stones with stone analysis at both time points. Yet, interpretation is limited as stone data for both time points were available in only 25.0% in the C group and 37.1% in the NC group.

While providing valuable insights our study has limitations, including the observational design, which may have introduced bias, as patients at higher stone risk may have been more likely to receive citrate. Furthermore, the restricted number of patients with UA and CaP stones limits generalizability of some findings. However, these limitations do not compromise the interpretation of our primary outcome, the potential metabolic side effects of citrate.

Our study has several strengths: (i) the large patient sample and extended FU period which provide robust data that strengthen the conclusions, (ii) the prospective design guaranteeing structured data collection, thereby minimizing recall bias, (iii) centralized urine analysis ensuring high accuracy and (iv) its foundation in a real-life cohort.

In conclusion, this is the first study focusing on adverse effects of citrate beyond 3 months of treatment. Chronic citrate was safe and not associated with long-term metabolic effects. However, due to potential confounding by indication, efficacy of treatment cannot be inferred from our study. Citrate treatment was linked to a greater reduction in RSR for UA, while the decrease of RSR for brushite was more pronounced in KSFs not treated with citrate. Despite a higher stone recurrence in citrate patients, most likely due to confounding by indication, we found no evidence for an increase in CaP stones. A prospective trial is needed to clarify the impact of repeated intensive nephrologist-led counseling with and without adjunctive citrate therapy, to determine the specific contribution of citrate to stone recurrence prevention.

## Supplementary Material

sfag058_Supplemental_File

## Data Availability

The data underlying this article will be shared on reasonable request to the corresponding author.
