# Supplementary material for: Long-term citrate treatment in high-risk kidney stone formers is not associated with metabolic adverse effects
Source: Clin Kidney J. 2026 Mar 9;19(5):sfag058. doi: 10.1093/ckj/sfag058 (PMC13176838; doi:10.1093/ckj/sfag058)
Supplement: sfag058_Supplemental_File [file sfag058_supplemental_file.zip › Supplemental_Figures_CKJ-01556-2025.pptx]

## Slide 1
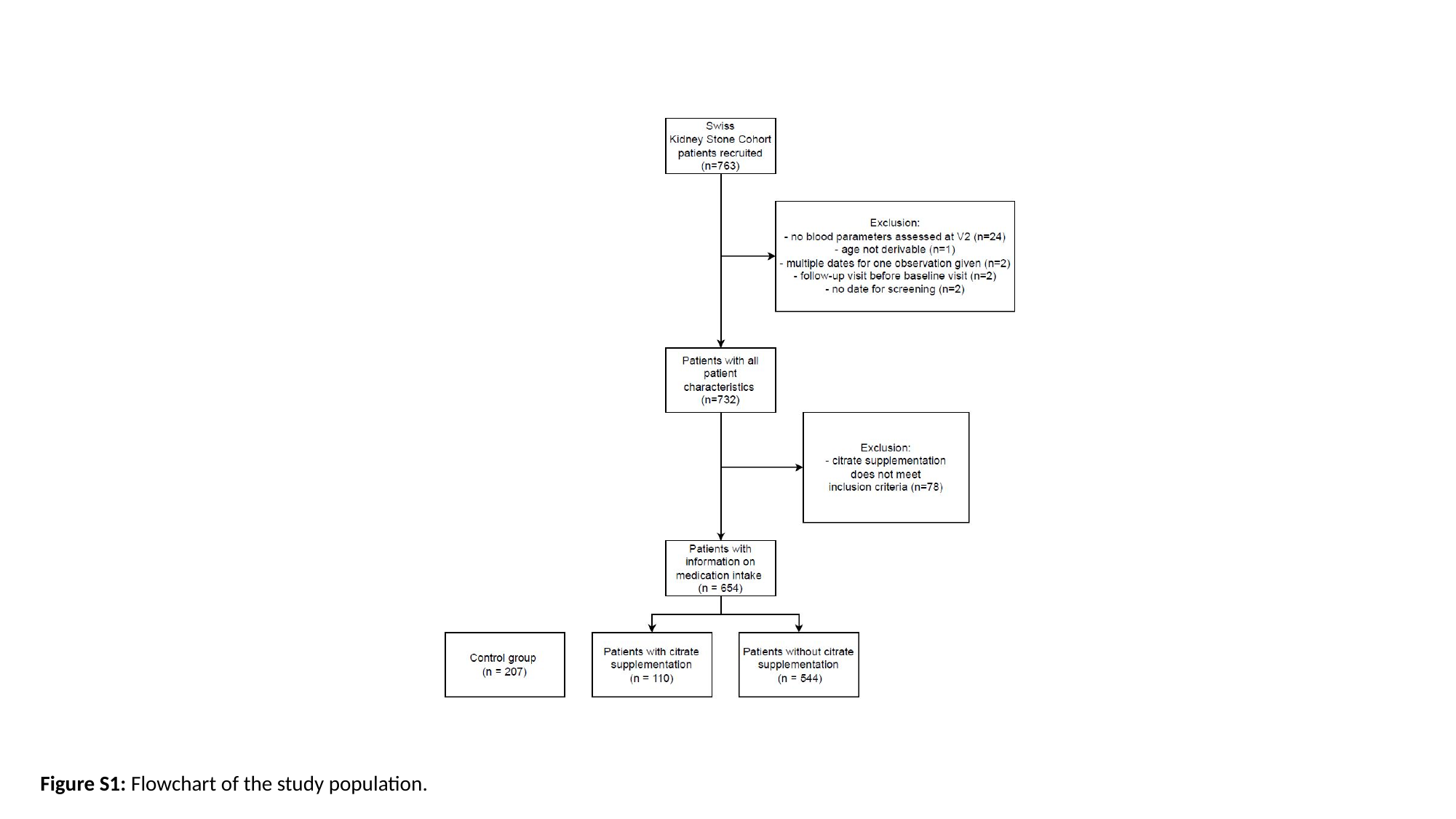

Figure S1: Flowchart of the study population.

## Slide 2
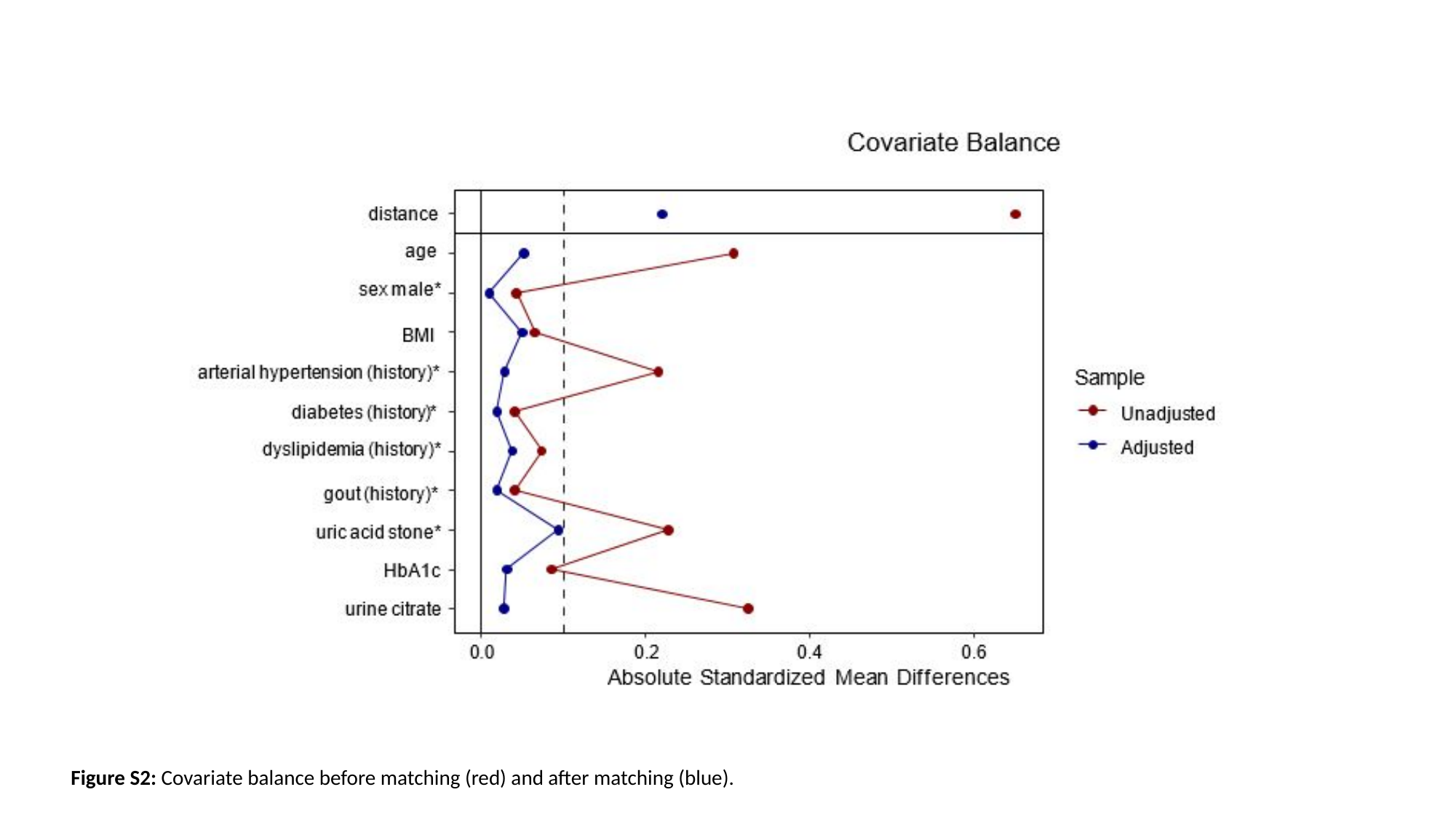

Figure S2: Covariate balance before matching (red) and after matching (blue).

## Slide 3
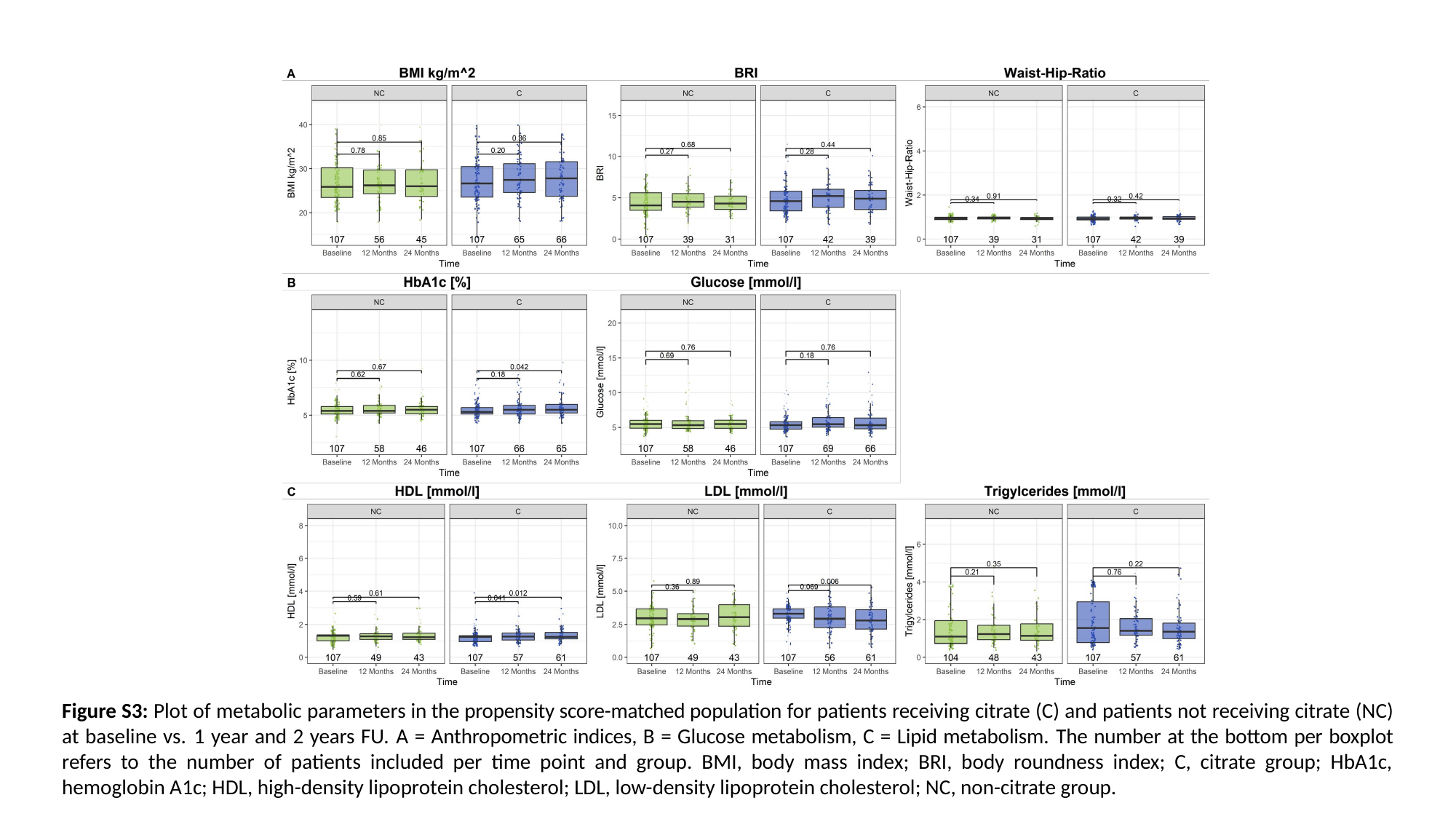

Figure S3: Plot of metabolic parameters in the propensity score-matched population for patients receiving citrate (C) and patients not receiving citrate (NC) at baseline vs. 1 year and 2 years FU. A = Anthropometric indices, B = Glucose metabolism, C = Lipid metabolism. The number at the bottom per boxplot refers to the number of patients included per time point and group. BMI, body mass index; BRI, body roundness index; C, citrate group; HbA1c, hemoglobin A1c; HDL, high-density lipoprotein cholesterol; LDL, low-density lipoprotein cholesterol; NC, non-citrate group.

## Slide 4
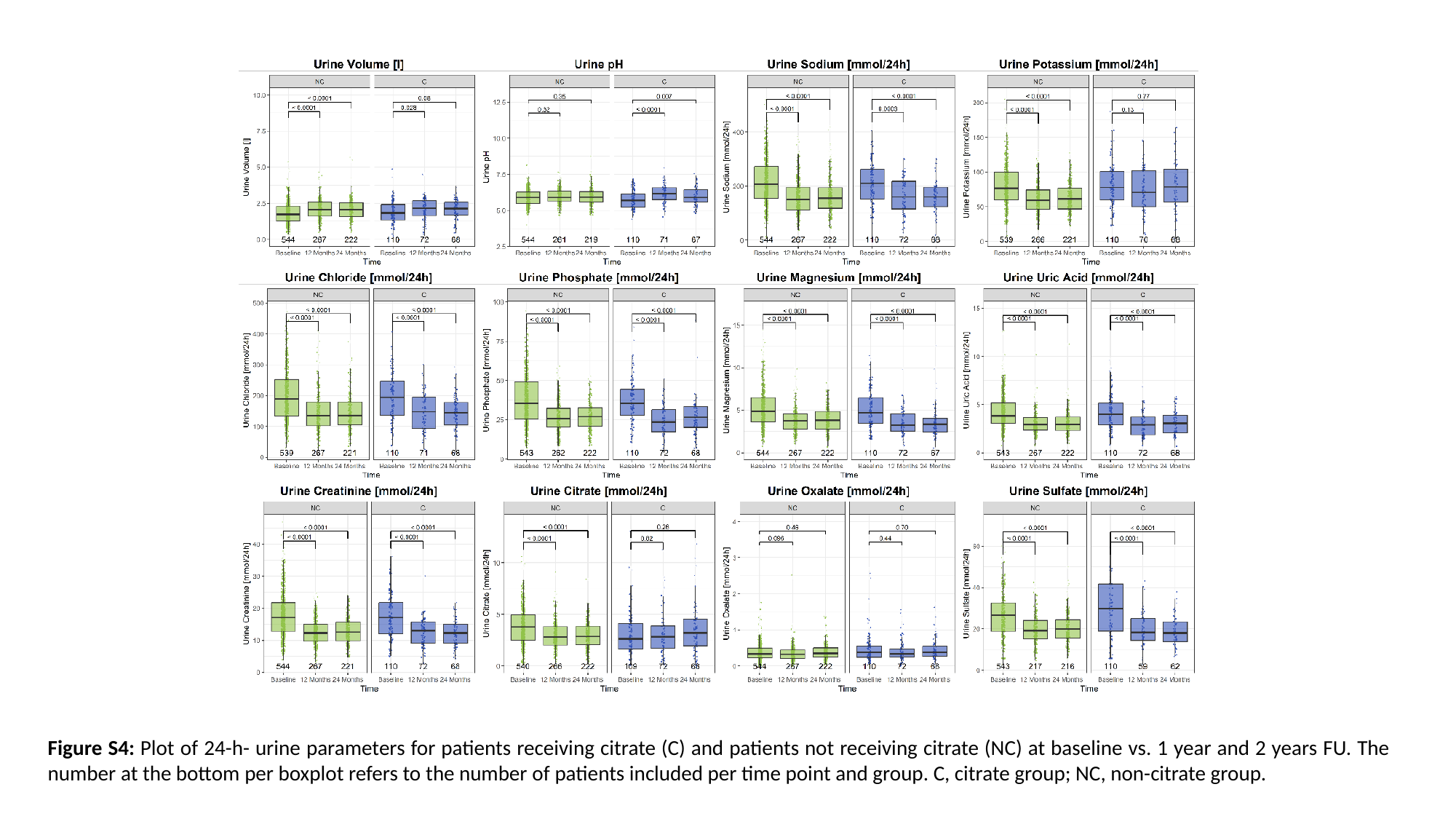

Figure S4: Plot of 24-h- urine parameters for patients receiving citrate (C) and patients not receiving citrate (NC) at baseline vs. 1 year and 2 years FU. The number at the bottom per boxplot refers to the number of patients included per time point and group. C, citrate group; NC, non-citrate group.

## Slide 5
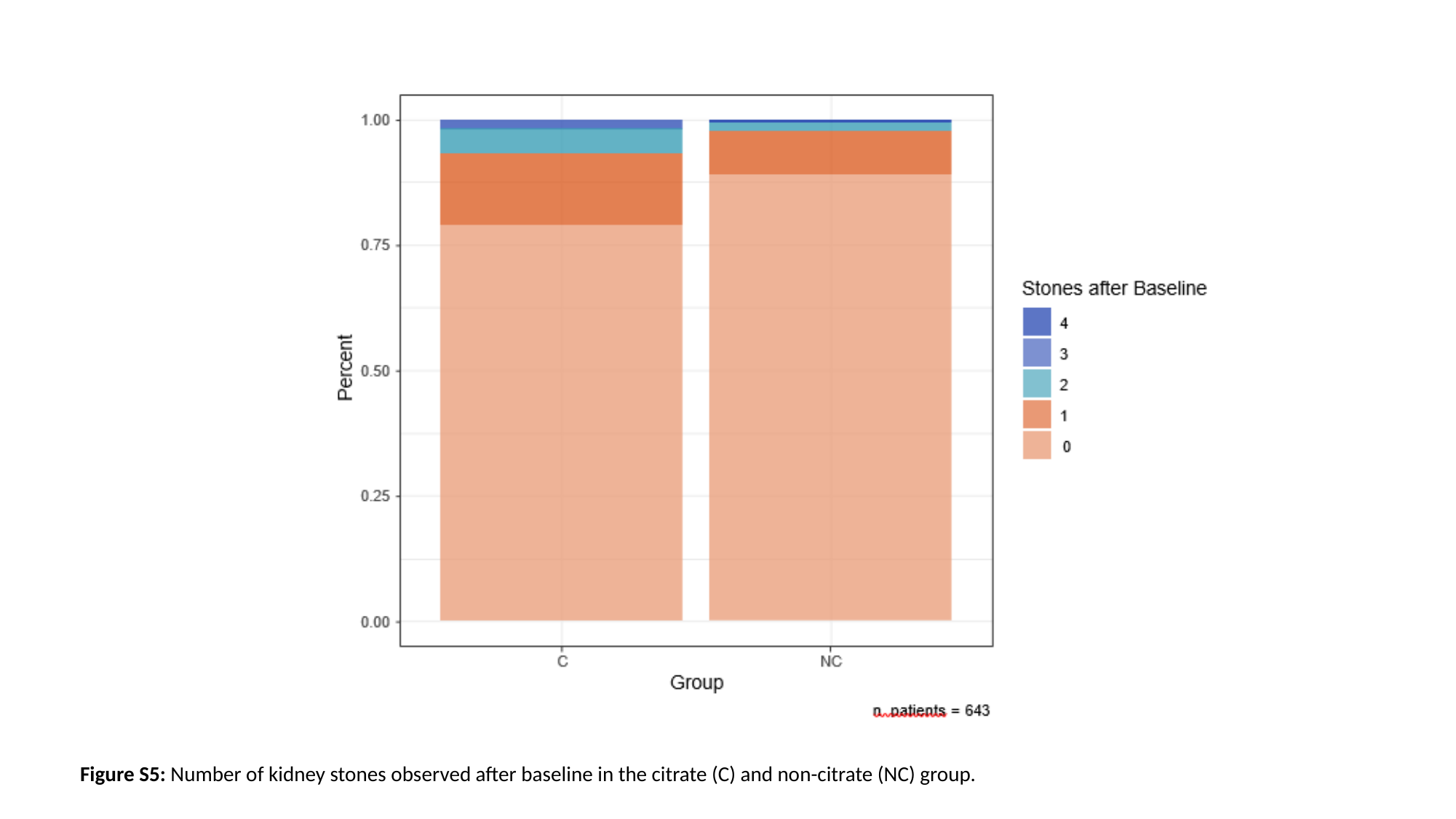

Figure S5: Number of kidney stones observed after baseline in the citrate (C) and non-citrate (NC) group.
